# Deletion of *A44L*, *A46R* and *C12L* Vaccinia Virus Genes from the MVA Genome Improved the Vector Immunogenicity by Modifying the Innate Immune Response Generating Enhanced and Optimized Specific T-Cell Responses

**DOI:** 10.3390/v8050139

**Published:** 2016-05-18

**Authors:** María Pía Holgado, Juliana Falivene, Cynthia Maeto, Micaela Amigo, María Fernanda Pascutti, María Belén Vecchione, Andrea Bruttomesso, Gabriela Calamante, María Paula del Médico-Zajac, María Magdalena Gherardi

**Affiliations:** 1 Instituto de Investigaciones Biomédicas en Retrovirus y SIDA (INBIRS), Universidad de Buenos Aires-CONICET, Facultad de Medicina, Ciudad de Buenos Aires 1121, Argentina; piaholgado@gmail.com (M.P.H.); juliana.falivene@gmail.com (J.F.); cynthiamayumi85@gmail.com (C.M.); mfernandezamigo.19@gmail.com (M.A.); fpascutti@yahoo.com.ar (M.F.P.); mb_vecchione@hotmail.com (M.B.V.); 2 Unidad de Microanálisis y Métodos Físicos Aplicados a Química Orgánica (UMYMFOR), Departamento de Química Orgánica, Facultad de Ciencias Exactas y Naturales, Universidad de Buenos Aires, Buenos Aires 1428, Argentina; andrea.bruttomesso@gmail.com; 3 Instituto de Biotecnología, CICVyA-INTA Castelar, Buenos Aires 1686, Argentina; calamante.gabriela@inta.gob.ar (G.C.); delmedicozajac.maria@inta.gob.ar (M.P.d.M.-Z.)

**Keywords:** MVA, vaccine, T-cell response

## Abstract

MVA is an attenuated vector that still retains immunomodulatory genes. We have previously reported its optimization after deleting the *C12L* gene, coding for the IL-18 binding-protein. Here, we analyzed the immunogenicity of MVA vectors harboring the simultaneous deletion of *A44L*, related to steroid synthesis and *A46R*, a TLR-signaling inhibitor (MVAΔA44L-A46R); or also including a deletion of *C12L* (MVAΔC12L/ΔA44L-A46R). The absence of biological activities of the deleted genes in the MVA vectors was demonstrated. Adaptive T-cell responses against VACV epitopes, evaluated in spleen and draining lymph-nodes of C57Bl/6 mice at acute/memory phases, were of higher magnitude in those animals that received deleted MVAs compared to MVAwt. MVAΔC12L/ΔA44L-A46R generated cellular specific memory responses of higher quality characterized by bifunctionality (CD107_a/b_^+^/IFN-γ^+^) and proliferation capacity. Deletion of selected genes from MVA generated innate immune responses with higher levels of determining cytokines related to T-cell response generation, such as IL-12, IFN-γ, as well as IL-1β and IFN-β. This study describes for the first time that simultaneous deletion of the *A44L*, *A46R* and *C12L* genes from MVA improved its immunogenicity by enhancing the host adaptive and innate immune responses, suggesting that this approach comprises an appropriate strategy to increase the MVA vaccine potential.

## 1. Introduction

Modified Vaccinia Ankara Virus (MVA) was originally developed as a vaccine for smallpox in primary chicken embryo fibroblasts (CEF) through serial passages of Chorioallantois Vaccinia virus Ankara (CVA). After this process, MVA lost its capacity to replicate *in vivo* in mammalian cells, restricting its productive replication to certain permissive cell-lines, such as BHK-21 [1,2,3]. A comparison of genomic sequences of CVA and MVA [4,5] indicated that, in addition to six large deletions, MVA presents mutations in more than 60% of the open reading frames (ORFs), however the mutations responsible of its *in vivo* attenuation remain unknown [6]. The MVA immunogenic capacity, as well as its high level of safety, and the feasibility to incorporate large foreign gene inserts has converted it to an attractive candidate for clinical vaccine development strategies against different infectious diseases such as HIV/AIDS, Malaria, Hepatitis B and C, Tuberculosis, and also smallpox [7,8,9,10,11,12,13,14,15,16]. Despite the loss of several immunomodulatory genes, MVA still preserves genes directed to evade the host immune response [17], the directed deletion from the MVA genome of these genes can constitute a strategy to improve its immunogenicity. This proof of concept was demonstrated previously, e.g., after deleting the gene encoding an interleukin-1β binding-protein [18], and also after the removal of the *A41L* gene encoding for a chemokine binding-protein [19]. Moreover, our group described that the deletion of the *C12L* gene from MVA genome, encoding for the interleukin-18 (IL-18) binding-protein generated a significant improvement in the immunogenicity of the vector leading to an increase in the magnitude and quality of specific cellular responses against Vaccinia (VACV) antigens and, more importantly, to HIV antigens [20]. Moreover, many other studies performed by other groups also demonstrated better levels of immunogenicity against recombinant antigens (mainly HIV proteins) expressed from the MVA specific deleted mutants [21,22,23,24].

The *A46R* VACV-gene still present in the MVA genome encodes for a protein that inhibits numerous TLR-signaling pathways through its binding to TIR domains [25] disrupting the association of adaptor proteins, such as MyD88, Mal/TIRAP, TRAM, and TRIF, consequently, prevents the interaction with the receptor and avoids the activation of NF-κB, IRF3/IRF7 and MAP-kinase pathways [26], thus contributing to the evasion of the immune response elicited by the host [27,28,29].

*A44L* is another VACV-gene present in the MVA genome encoding a 3β-hydroxysteroid-dehydrogenase/∆5-∆4 isomerase (3β-HSD), which participates in steroid hormone metabolism, catalyzing reactions like the conversion of pregnenolone into progesterone [30], among others. Some steroid hormones are glucocorticoids considered to be potent immunosuppressive and anti-inflammatory agents that modulate cytokine production, and the migration and cytotoxicity of immune cells [31].

Previous reports have demonstrated that deletion of *A46R* from the VACV genome (Western Reserve strain, WR) generated an attenuated intranasal infection in a murine model [26] and that after its deletion from the New York Vaccinia Virus (NYVAC) genome backbone, immune responses against recombinant HIV antigens were improved [32]. However, previous reports describing its deletion from the MVA genome indicated the failure to improve vector immunogenicity [33,34].

In relation to the *A44L* gene, previous data showed that, in the WR strain, it contributes to virulence after intranasal [35,36] or intradermal infection of mice [37], and also that its deletion produced an increase in the inflammatory response and cytotoxic T lymphocyte activities after intranasal inoculation of mice [30]. However, previous studies described that the deletion of this gene from MVA generated no increase in its immunogenicity [33,34].

In this study we did an in-depth characterization of the effects of simultaneous deletion of the *A46R* and *A44L* genes from the MVA genome. For this, we deleted the *A44L-A46R* segment that also includes the *A45R* gene. However, as the product of this last gene is an inactive superoxide dismutase-like protein [4,38], we did not focus on the effect of *A45R* absence. Importantly, in contrast with other studies, we demonstrated for each of the immunomodulatory viral genes that their removal from the vector genome generated an ablation of their biological functions. Then, in an *in vivo* mouse model, we showed that by deleting the *A46R* and *A44L* genes from MVA, in combination with the *C12L* gene, improves the immunogenicity of the vector inducing an immune response of higher magnitude and better quality in comparison with the MVAwt. Moreover, we found that the deletion of these three viral genes produced an increase in the *in vivo* inflammatory innate immune responses which have an impact on the generation of adaptive immune responses.

## 2. Materials and Methods

### 2.1. Cells and Viruses

MVA-deleted viruses were generated in primary cultures of chicken embryo fibroblasts (CEFs) as described previously [20].

BHK-21 (ATCC CCL-10) and BSC-40 (ATCC CRL-2761) were grown in Dulbecco’s Modified Eagle’s Medium (DMEM) and THP-1 (ATCC TIB-202) cells in RPMI-1640, both supplemented with 2 mM l-glutamine, penicillin 100 U/mL, streptomycin 0.1 mg/mL (DMEMc and RPMIc, respectively) and complemented with 10% heat inactivated fetal bovine serum (FBS). Cells were maintained at 37 °C in a 5% CO_2_ atmosphere. THP-1–derived macrophages were obtained by stimulating THP-1 monocytes with 50 ng/mL of phorbol-12-myristate-13-acetate (PMA, Sigma-Aldrich, St. Louis, MO, USA) for three days.

### 2.2. Construction of Deleted MVAs: MVA∆A44L-A46R and MVA∆C12L/∆A44L-A46R

To construct an MVA lacking the *A44L* and the *A46R* VACV genes, *A44L-*gene upstream-region and *A46R*-gene downstream-regions (nucleotide positions 142215–142569 and 144651–144994, respectively, GeneBank sequence ID: U94848.1) were amplified by PCR and cloned into pBlueScript (Stratagene) obtaining the intermediate plasmid pBS-A44L-A46R. Primers used were 157iF 5′-AGAGCTCGGATAAAATCAAAATTACGGTTG-3′/157iR 5′-AGCGGCCGCCGAACACGGAAATGGCTAGA-3′ and 159dF: 5′-CGGATCCGATGATTCGTCTACATGTTC-3′/159dR: 5′ AGAATTCGCCCACATAAATGCGTTGGAG-3′. Then, the *uidA* gene (β-glucuronidase enzyme, GUS) under the poxviral H6-promoter (available in our laboratory) was subcloned into pBS-A44L-A46R obtaining p∆A44L-A46R (transference-vector). Identities of all plasmids were verified by DNA sequencing. The p∆A44L-A46R plasmid was transfected into cells infected with the MVA-F6 isolate obtained after 582 passages in CEF cells [39] provided by G. Sutter (Paul-Ehrlich-Institut, Langen, Germany), or with the MVA∆C12L vector [20]. MVA∆A44L-A46R and MVA∆C12L/∆A44L-A46R were wild-type virus-free after ten or six consecutive rounds of plaque purification on CEFs in the presence of GUS substrate (X-gluc, Inalco, San Luis Obispo, CA, USA), respectively. Purity of the selected clones was verified by PCR, employing specific primers.

#### RT-PCRs

CEFs were non-infected or infected at multiplicity of infection (moi) of 1 with MVAwt, MVA∆A44L-A46R or MVA∆C12L/∆A44L-A46R. Twenty-four hours post-infection (hpi) cells were harvested and RNA was extracted using Trizol following the manufacturer´s instructions (MRC Inc, Cincinnati, OH, USA). RNA samples were treated with DNaseI (Invitrogen, Vilnius, Lithuania) and reverse-transcribed with M-MLV reverse-transcriptase (Promega, Madison, WI, USA) using random hexamers. The *A44L*, *A46R*, *C12L*, and *TK* (thymidine-kinase) genes were amplified from cDNA. Primers used were for *A44L*: 159intF 5′-CTTAAAGTAGACTCCGATTC-3′/159STOP5′-TTATACATCCGTTTCCCTG-3′, for *A46R*: 157intF 5′-GGAAATGCTTACTTTTGCTA-3′/157STOP 5′-TTATTCTGATTCTTCTAGCC-3′, for *C12L* detailed in [20] and for *TK*: TK1 5′-TCCCCGCGGTGAACGGCGGACATATTC-3´/TK4 5′-GGGGTACCTTATGAGCCGACGTAACA-3′.

### 2.3. Viral Immunization Stocks

MVA viral stocks were grown in BHK-21 cells, purified by ultracentrifugation through 45% sucrose-cushion and titrated by immunostaining using a rabbit polyclonal antibody against B5 and A33 VACV-proteins (#V0500-11D, US Biological, Salem, MA, USA) [40].

#### *In Vitro* Characterization of MVA∆A44L-A46R and MVA∆C12L/∆A44L-A46R Viruses

To analyze the virus growth kinetics, BHK-21 monolayers (70% confluence) were infected (moi = 0.01) with different MVAs. For each virus and time point, cells (intracellular-virus) and supernatants (extracellular-virus) were collected separately, frozen/thawed three times, and stored at −80 °C until titration by immunostaining. Each time point was evaluated by duplicate.

### 2.4. THP-1 Infection and Cytokine Production Evaluation

THP-1–derived macrophages were uninfected or infected with distinct MVAs (moi = 5). At different hours post-infection supernatant was collected and stored at −80 °C for future analysis. IL-1β was measured with a commercial kit (Biolegend, San Diego, CA, USA) following the manufacturer’s instructions.

### 2.5. 3β-HSD Activity

We followed the protocol described by Moore and Smith [35] with slight modifications. Briefly, BSC-40 cells were uninfected or infected with distinct MVAs (moi = 10), 18 h later cells were washed and incubated for 1 h at 37 °C with Buffer A [35], 0.66 mM β-nicotinamide adenine dinucleotide (NAD, Sigma, St Louis, MO, USA) and 4 mM 5-pregnen-3β-ol-20-one (Sigma, St Louis, MO, USA). Afterwards, cells were permeabilized with 1% saponin for 1 h at 37 °C and the reaction was stopped with 1.2 N HCl. Subsequently, we added 10 nmol of 5-pregnen-16-methylene-3β,17-diol-20-one3-acetate as an internal control, in order to check the efficiency of the process. Extraction of steroids was performed with ethyl acetate, subsequently evaporated, and then methanol-dissolved steroids were analyzed by LC-MS/MS.

### 2.6. IL-18 Binding-Protein Bioassay

The functional assay to evaluate the effect of the absence of the C12 protein in these new deleted vectors was performed as described previously by Falivene *et al.* [20] with some modifications. Briefly, BHK-21 cells instead of CEFs, were uninfected or infected with distinct MVAs (moi = 5). Twenty-four hours post-infection, supernatants were harvested, clarified from cell debris and viral particles, and stored at −20 °C until use. Splenocytes from C57Bl/6 instead of Balb/C mice were cultured in RPMIc plus 10% of FBS with 200 ng/mL Concanavalin A (ConA) alone or in combination with 10 ng/mL of murine recombinant IL-18 (rIL-18), for 24 h at 37 °C. To evaluate the inhibitory action of C12 on IL-18, the rIL-18 was pre-incubated with clarified supernatants defined before, for 1 h at room temperature. After that, levels of IFN-γ in culture supernatants were measured by IFN-γ ELISA set, following the manufacturer’s instructions (BD Biosciences, San Diego, CA, USA).

### 2.7. Immunization Protocols, Sample Collection, and Processing

SPF C57Bl/6 (H-2^b^) female mice, 6–8 weeks old, were purchased from the laboratories of the School of Veterinary Sciences, University of La Plata, Buenos Aires, and housed in our animal facilities. All experiments were carried out in accordance with recommendations in the Guide for the Care and Use of Laboratory Animals (NIH). The protocol was approved by Committee of Care and Use of Laboratory Animals from School of Medicine, University of Buenos Aires (Permit Number: 508/2009). All immunizations were performed intramuscularly (i.m) in 100 µL of PBS (50 µL per leg, in the tibial muscle), with a dose of 1 × 10^7^ plaque-forming units (PFU) of the indicated virus. Seven or 45 days after immunization, mice were sacrificed to collect samples (spleen and inguinal lymph nodes, ILNs). Blood samples were obtained by cardiac punction from anesthetized mice and sera were obtained by standard procedures. Splenocytes and lymphocytes from ILNs were isolated by routine methods [41].

### 2.8. Analysis of Specific T-Cell Immune Responses

#### 2.8.1. Peptides

Lyophilized VACV-specific synthetic peptides (JPT Peptide Technologies) dissolved in dimethylsulfoxide (DMSO, Sigma-Aldrich, St Louis, MO, USA) were used at a final concentration of 2 μg/mL for ELISPOT and ELISA assays, and 4 μg/mL for flow-cytometry assays. B8R and E9L, H3L, and L4R peptides were previously described as MHCI restricted and as MHCII restricted epitopes in C57Bl/6 mice (CD8^+^ and CD4^+^ specific, respectively) [42].

#### 2.8.2. Murine IFN-γ and IL-2 ELISPOT Assays

ELISPOT assays were performed using freshly isolated splenocytes and cells from ILNs as described previously [43]. Briefly, 5 × 10^4^ to 10^6^ cells in RPMIc were plated in triplicate on nitrocellulose 96-well plates (Millipore, Billerica, MA, USA) coated with anti-mouse IFN-γ or anti-mouse IL-2 antibodies (BD Biosciences). Specific stimuli consisted in VACV-specific peptides, RPMIc with 0.08% of DMSO (negative control) and concanavalin A (ConA 1 mg/mL, positive control). The response was considered positive when the number of specific spots per well overcame at least two times the average from negative control of each group.

#### 2.8.3. Intracellular Cytokine Staining (ICS) and Proliferation Assays

Splenocytes were dispensed in 96-well U bottom plates (10^6^ cells/well) and stimulated with B8R (4 μg/mL) for 5 h in presence of co-stimulatory antibody anti-CD28 (1 ng/mL), brefeldin A (1 μL/mL GolgiPlug), monensin (0.7 μL/mL GolgiStop), and monoclonal antibodies anti-CD107_a_-FITC and anti-CD107_b_-FITC (all reagents from BD Biosciences) [44]. Negative and positive controls consisted of cells stimulated with RPMIc plus 0.16% of DMSO, or PMA/Ionomycin (PMA 10 ng/mL plus ionomycin 250 ng/mL, Sigma-Aldrich), respectively. Afterwards, cells were washed and stained with a probe to distinguish between live and dead cells (Live/Dead reactive, Life Technologies, Burlington, ON, Canada) and with surface antibodies: CD8-PerCP or CD4-PerCP, CD44-PECy7, CD62L-APC for 30 min at 4 °C. After that, cells were permeabilized and fixed using Cytofix/Cytoperm kit, stained using anti-IFN-γ-PE (all reagents from BD Biosciences) for 30 min at 4 °C in the dark, washed and acquired in a FACSCanto flow cytometer (BD Biosciences).

Proliferation assays were performed as we described previously [45,46]. After labeling with carboxyfluorescein-succinimidyl-ester (CFSE), cells were cultured in 12-wells plates (10^7^ cells/mL) and stimulated with UV-inactivated VACV (2.5 μg/mL), PMA/Ionomycin (positive controls) or with medium alone (negative controls). After four days, lymphocytes were harvested, counted, and washed with RPMIc. Then, cells were stained for 30 min at 4 °C with anti-mouse CD8-PerCP or CD4-PerCP, CD44-PECy7, CD62L-APC (BD Biosciences) plus the viability dye, washed, and acquired in a FACSCanto flow cytometer.

Data acquisition and analysis were performed with FACSDiva Software (BD Biosciences). Instrument settings and fluorescence compensation were performed on each testing day using unstained and single-stained samples. Stimulated cells stained for surface molecules and isotype matched controls were included in each experiment.

#### 2.8.4. T-Cell Specific Cytokine Production

Splenocytes were suspended in RPMIc, cultured as we explained in [45] and stimulated for 72 h with the indicated peptides, ConA or RPMIc. Then, supernatants were harvested and stored at −80 °C until use. Cytokine production was evaluated using IFN-γ and IL-2 ELISA sets (BD Biosciences) or the Th1/Th2 cytokine kit (Cytometric Bead Array, CBA, BD Biosciences), following the manufacturer's instructions. The response was considered positive when cytokine quantities exceeded the average values found in negative controls of each group plus 3 SD.

#### 2.8.5. *In Vitro* Cytotoxicity Assays

The *in vitro* cytotoxicity assay was based on Nakagawa *et al.* [47]. Splenocytes from MVAwt or MVA∆C12L/∆A44L-A46R immunized mice (7 dpi) were used as effector cells (cytotoxic T-CD8^+^ cells). Splenocytes from *naïve* C57Bl/6 mice were used as target cells. For that, target cells were stained with 6 uM of CFSE (control target) or 16 uM (sensitive target). Sensitive target cells were pulsed with B8R peptide (5 μg/mL) for 30 min at 37 °C. For sensitive reactions, effector cells (1 × 10^5^ cells) were incubated with different amounts of sensitive target cells for 4 h at 37 °C. The ratio of Effector/Target cells was 0.5:1 to 10:1. In the same way, control target cells were incubated with effector cells, covering the same ratio. After incubation, sensitive and control target cells at the same Effector/Target ratio were mixed, washed with PBS and the acquisition of the cells was performed using a BD FACSCanto flow cytometer.

### 2.9. Innate Immune Response Analyses

Groups of four to six C57Bl/6 mice were i.m immunized with 2 × 10^7^ PFU of MVAwt or MVAΔC12L/ΔA44L-A46R (100 μL, 50 μL per leg in tibial muscle). At each time point, animals were sacrificed and blood and ILNs were obtained by routine methods. For cytokine analysis, ILNs of each mouse were suspended in PBS plus Protease Inhibitor and EDTA (Pierce) processed with a homogenizer (UltraTurrax-T25 homogenizer, IKA, Staufen, Germany) and transferred immediately to ice. Samples were centrifuged and supernatants were stored at −80 °C until analyzed. Cytokines were measured by ELISA (anti-mouse ELISA sets for IFN-γ, IL-12 and IL-1β, BD Pharmigen, San Diego, CA, USA).

RNA extraction was performed using the TRIzol reagent and the Pure Link RNA Mini Kit (Ambion, Burlington, ON, Canada). RNA (5 μg) was treated with DNAse I and reverse-transcribed as we described before. Quantitative PCR was performed with a 7500 Real-Time PCR System (Applied Biosystems, Foster City, CA, USA) using SYBR Select Master Mix (Life Technologies) and the following primers: IFN-βF 5′-GCACTGGGTGGAATGAGACT-3′/IFN-βR 5′-AGTGGAGAGCAGTTGAGGACA-3′, β-actinF5′-TGTCCACCTTCCAGCAGATGT-3′/β-actinR5′-AGCTCAGTAACAGTCCGCCTAGA-3′. All samples were tested in triplicates. Amplifications consisted in a denaturation step (95 °C, 15 s) and an annealing/extension step (60 °C, 1 min). IFN-β expression was normalized to β-actin expression in arbitrary units (A.U.), and then all groups were relativized to the naïve group.

### 2.10. Data Analysis

Statistics were performed using the GraphPad Prism [48]. In all cases background values (negative control) were subtracted. Statistical tests employed were specified in each assay.

## 3. Results

### 3.1. In Vitro Characterization of MVA Vectors after Deleting A44L, A46R, and C12L Genes

The removal of the *A44L*, *A46R* and *C12L* genes from deleted MVAs was firstly corroborated by specific PCR (see supplemental data Figure S1).

Afterwards, in order to verify the absence of mRNA expression of the different genes, we performed RT-PCRs with RNA extracted from CEFs previously infected with parental (MVAwt) or the deleted viruses (MVA∆A44L-A46R and MVA∆C12L/∆A44L-A46R), using specific primers detailed in Materials and Methods.

**Figure 1:**
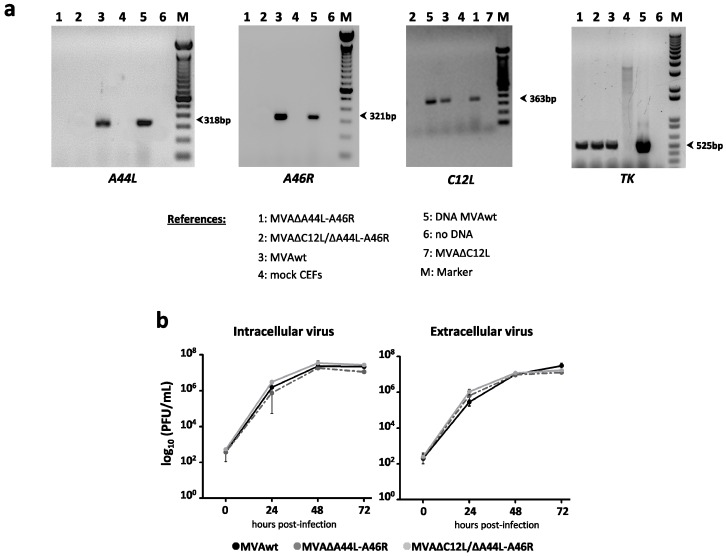
*In vitro* characterization of MVA∆A44L-A46R and MVA∆C12L/∆A44L-A46R. (**a**) RNA was extracted from mock or infected CEFs with the different MVAs at 24 hpi (moi = 1), specific mRNAs were assessed by RT-PCR; and (**b**) virus growth kinetics of MVA vectors. BHK-21 were infected with the indicated MVA at moi = 0.01. At different hpi virus production was titrated by immunostaining.

Figure 1a shows the amplification of *A44L* and *A46R* mRNAs (318 bp and 321 bp, respectively), only present in MVAwt infected cells, and the amplification of *C12L* mRNA (363 bp), present in MVAwt and MVA∆A44L-A46R but not in MVA∆C12L/∆A44L-A46R infected cells. Thymidine kinase mRNA (TK, 525 bp) was used as positive control of viral mRNA amplification.

Afterwards, we analyzed the replicative efficiency of the vectors performing multiple-step growth curves for all MVAs, corroborating that MVA∆A44L-A46R and MVA∆C12L/∆A44L-A46R did not differ in their growth kinetics curve from MVAwt (Figure 1b). Therefore, *A44L*, *A46R*, and *C12L* genes are not essential for MVA replication in cell culture, even if they are simultaneously deleted.

In order to evaluate if the MVA vectors showed a similar capacity to infect antigen-presenting cells, the human monocytic cell-line THP-1 was infected with the different MVAs and 24 hpi the presence of early/late VACV proteins (B5R and A33R) was analyzed by immunofluorescence [49,50]. In Figure S1 it can be seen that all MVA vectors have similar and comparable capacities to infect THP-1 cells, showing an equivalent intracellular localization of the viral proteins.

### 3.2. In Vitro Analysis of the Biological Effects of Deleted Viral Genes

**Figure 2:**
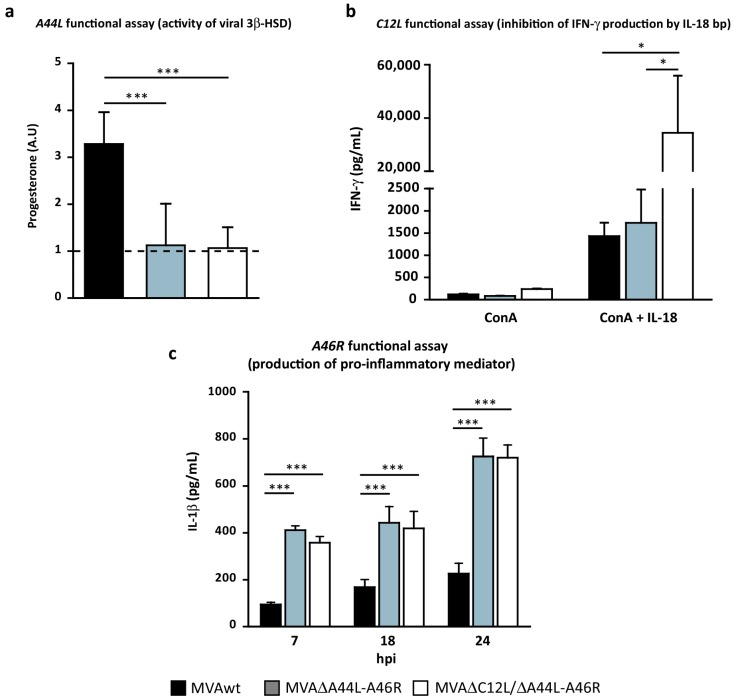
Functional assays of selected genes *A44L*, *C12L*, and *A46R*. (**a**) *In vitro* progesterone production after infection of BSC-40 with the indicated virus. Progesterone was normalized to an internal control (to check extraction efficiency of steroids) and then related to mock levels (dotted-line). Mann-Whitney test; (**b**) Splenocytes from naïve mice treated with ConA (0.2 ug/mL) alone or combined with rIL-18 (10 ug/mL) were incubated with supernatants (virus-free) from BHKs infected with the indicated MVAs. IFN-γ was quantified in splenocyte-culture supernatants by ELISA after 24 h; (**c**) *In vitro* quantification by ELISA of pro-inflammatory mediator in supernatants of THP-1 after infection with distinct MVAs. Two-way ANOVA and Bonferroni’s post-test. Statistically significant differences: * *p <* 0.05, *** *p <* 0.001.

Once we confirmed the absence of expression of the *A46R*, *A44L*, and *C12L* genes from the deleted MVAs, we considered it highly relevant to evaluate the biological impact that these deletions could have. The biological function of *A44L* (equivalent to progesterone production by 3β-HSD enzyme) was assayed as described in the methodology. In Figure 2a it can be clearly seen that in MVAwt infected cells, progesterone levels were significantly higher than those found in MVA∆A44L-A46R or MVA∆C12L/∆A44L-A46R infected cells, which were similar to those observed in mock-infected cells, indicating that the viral ability to synthesize progesterone was no longer present in deleted MVAs.

In order to analyze the ability of the C12 protein to inhibit the biological activity of mouse IL-18, we treated splenocytes from C57Bl/6 naïve mice with ConA or ConA plus rIL-18 in the presence of supernatants from MVA infected BHK-21 cells, as explained in Materials and Methods. Twenty-four hours later, IFN-γ was quantified in supernatants of splenocyte-cultures. Significantly higher levels of IFN-γ were found in splenocyte-cultures activated with ConA plus rIL-18 incubated with supernatants from MVA∆C12L/∆A44L-A46R, in comparison to those incubated with supernatants from MVAwt or MVA∆A44L-A46R (Figure 2b), indicating that IL-18 binding-protein was no longer present in cells infected with MVA∆C12L/∆A44L-A46R.

Next, in order to evaluate the consequences of *A46R* absence, we infected THP-1 cells and evaluated IL-1β production as explained in Materials and Methods. Since A46 has been characterized as an antagonist of host IL-1 and TLR-signaling [26], quantifying the IL-1β secreted after activation of these pathways, by MVA infection, might be a way to measure the biological effect of presence/absence of A46. As it can be appreciated in Figure 2c we found significant higher levels of IL-1β in cells infected with MVA∆A44L-A46R and MVA∆C12L/∆A44L-A46R vectors in comparison to MVAwt.

The results described in this section indicated that in both deleted MVAs, the biological functions of the *A44L*, *A46R*, and *C12L* genes (as anti-host immune response modulators) were absent at least in the *in vitro* models assayed.

### 3.3. Modulation of Specific T-Cell Responses by MVA∆A44L-A46R and MVA∆C12L/∆A44L-A46R Vectors

**Figure 3:**
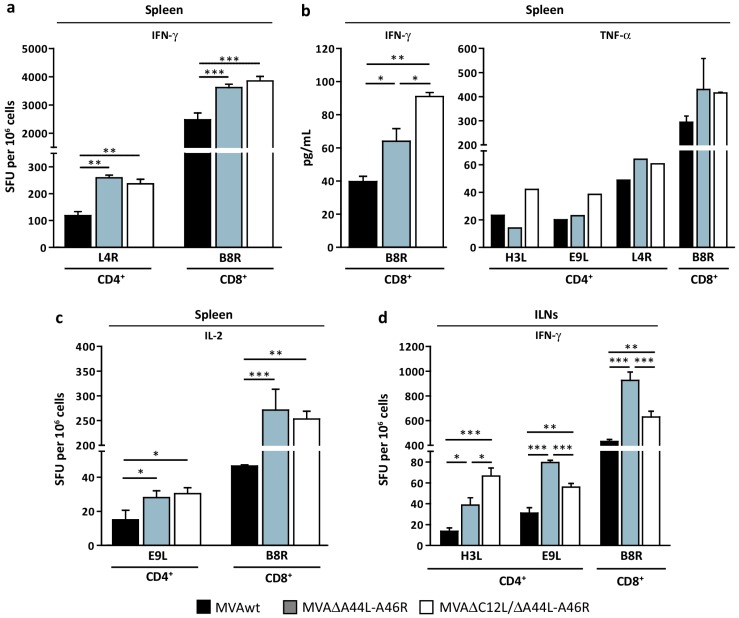
Modulation of specific T-cell response after VACV gene deletion. Groups of four C57Bl/6 mice were i.m immunized and 7 dpi specific T-cell responses were evaluated in spleen (**a**–**c**) and ILNs (**d**). Magnitude of the response was measured by IFN-γ (**a**,**d**) and IL-2 (**c**) ELISPOT assays. Specific cytokine production in splenocyte-culture supernatants was evaluated by ELISA (**b**, left panel) or CBA (**b**, right panel) after stimulation with indicated VACV-peptides. Data are expressed as mean + SD of triplicate (**a**–**d**) or duplicate (**b**) determinations, and are representative of three independent experiments. Statistical analysis performed by one-way ANOVA and Bonferroni’s post-test. Significant differences: * *p <* 0.05, ** *p <* 0.01, *** *p <* 0.001. SFU: Spot-forming unit.

After the *in vitro* characterization of the new deleted MVA vectors described above our next aims were focused in the study of the effects caused by removing the selected genes from the MVA genome on its immunogenicity potential. For this, we immunized groups of four C57Bl/6 mice by intramuscular (i.m) route with 10^7^ PFU of the different MVAs, and seven days post-inoculation (dpi) the anti-viral specific cellular immune response induced was evaluated against VACV-epitopes. Figure 3 shows the specific anti-VACV response elicited by the deleted MVAs compared to MVAwt. We found that both deleted MVAs elicited a higher response (IFN-γ secreting-cells) in comparison with MVAwt for both CD4^+^ (L4R) (*p <* 0.01) and CD8^+^ (B8R) (*p <* 0.001) T-cell epitopes (Figure 3a).

In concordance, we also observed a significant increase in IL-2 secreting-cells against E9L (CD4^+^ epitope) (*p <* 0.05) and B8R (CD8^+^ epitope) peptides (*p <* 0.001 MVA∆A44L-A46R *vs.* MVAwt; *p <* 0.01 MVA∆C12L/∆A44L-A46R *vs.* MVAwt) (Figure 3c). Even more, analysis of cytokine production by splenocytes of the distinct groups, after stimulation with specific VACV-peptides, showed that cells from mice immunized with MVA∆A44L-A46R or MVA∆C12L/∆A44L-A46R produced 1.5 or up to 2-fold higher levels of IFN-γ than MVAwt (*p <* 0.05 and *p <* 0.01 respectively), and a tendency to secrete higher TNF-α amounts against the different peptides (Figure 3b).

Considering the importance that draining lymph nodes (LNs) to the site of inoculation have in the final outcome of the immune response [51] we also analyzed T-cell responses in inguinal LNs (ILNs) (Figure 3d). We found that both deleted MVAs elicited a higher specific response against CD4^+^ and CD8^+^ VACV-peptides compared to MVAwt. Additionally, the response generated by MVA∆A44L-A46R was significantly higher than MVA∆C12L/∆A44L-A46R (*p <* 0.001 for B8R and E9L). These results are in line with those observed in spleen and both indicated that specific T-cell responses against VACV at 7 dpi were increased in mice immunized with deleted MVAs in comparison to mice that received MVAwt.

### 3.4. At Later Times Post-Immunization Deleted MVAs Still Induced Higher Specific T-Cellular Responses

**Figure 4:**
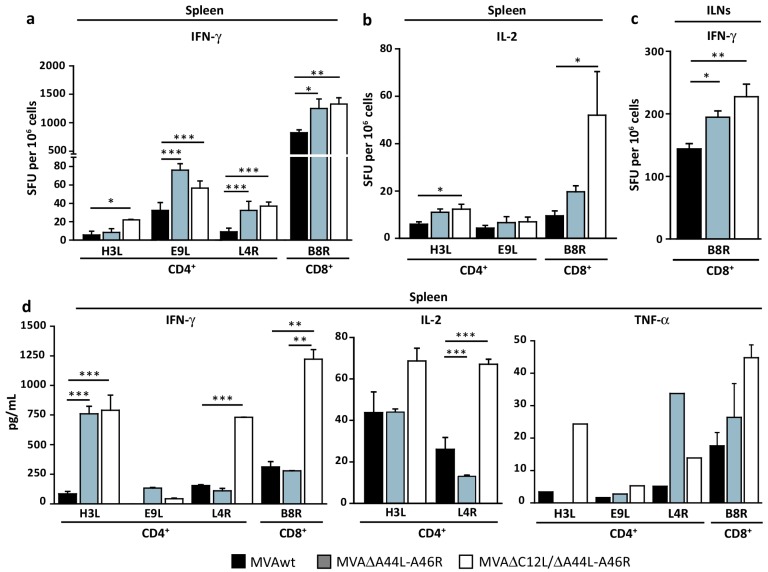
Deletion of specific VACV genes improved adaptive T-cell response during memory phase. Groups of four C57Bl/6 mice were immunized and 45 dpi specific T-cell responses against VACV-peptides were evaluated in spleen (**a**,**b**) and ILNs (**c**). Specific responses were evaluated by IFN-γ (**a**,**c**) and IL-2 (**b**) ELISPOT. Specific cytokine production in splenocyte-culture supernatants was evaluated by ELISA (**d**, left and middle panel) or CBA (**d**, right panel) after stimulation with VACV-peptides. Data are expressed as mean + SD of triplicate (**a**–**c**) or duplicate (**d**) determinations, and are representative of three independent experiments. Statistical analysis performed by one-way ANOVA and Bonferroni’s post-test. Significant differences: * *p <* 0.05, ** *p <* 0.01, *** *p <* 0.001.

The specific memory cells induced after vaccination are essential players in the host defense after the reintroduction of a pathogen antigen. To evaluate this, immune responses were studied 45 dpi in mice immunized with the different MVAs in spleen (Figure 4a,b,d) and ILNs (Figure 4c).

Both deleted MVAs induced higher magnitudes of anti-VACV IFN-γ secreting-cells than MVAwt (Figure 4a,c). Importantly, MVA∆C12L/∆A44L-A46R elicited higher numbers of IL-2 secreting CD4^+^ and CD8^+^ T-cells than MVAwt (Figure 4b). This pattern of responses were corroborated by quantifying IFN-γ, IL-2, and TNF-α in supernatants of splenocytes from different mice groups re-stimulated with VACV-peptides (Figure 4d), where differences between MVAwt and MVA∆C12L/∆A44L-A46R were even amplified.

It must be mentioned that we had also quantified IL-4 and IL-5 (Th2 cytokines) in supernatants of splenocytes re-stimulated with the different VACV-peptides but, in all cases, cytokines levels were under the limit of detection of the technique employed. Additionally, we analyzed antibody responses induced by deleted MVAs, by quantifying IgG levels in serum from immunized mice. Although we found slight differences when pooled serum samples were analyzed, in individual sera we did not find significant increases in antibody responses in animals immunized with deleted MVAs, at least for antibodies directed against surface VACV-epitopes (see supplemental data Figure S2).

### 3.5. MVA∆A44L-A46R and MVA∆C12L/∆A44L-A46R Vectors Improved the Quality of Memory T-Cell Responses

**Figure 5:**
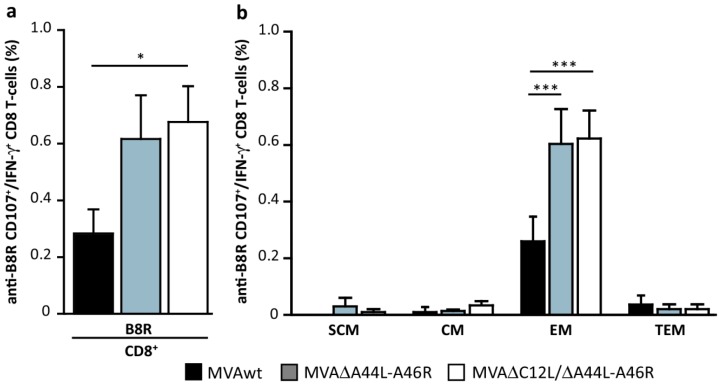
Modulation of specific responses in memory T-cell subpopulations. (**a**) Forty five days after immunization, splenocytes from immunized mice, were stimulated with B8R peptide. Percentage of double-positive (CD107_a/b_^+^_/_IFN-γ^+^) CD8^+^T-cells was analyzed by flow-cytometry. (**b**) Distribution of memory-subpopulations among bifunctional B8R-specific T-cells, classified as follows: ‘‘early-memory or T stem-cells memory’’ (T_SCM_; CD44L^−^/CD62L^+^), T central memory (T_CM_; CD44^+^/CD62L^+^), T effector memory (T_EM_; CD44^+^/CD62L^−^), and T terminal effector memory (T_TEM_; CD44^−^/CD62L^−^). Data are representative of three independent experiments. Statistical analysis performed by one-way ANOVA and Bonferroni’s post-test. Significant differences: * *p <* 0.05, *** *p <* 0.001.

Subsequently, we evaluated the quality of the memory CD8^+^T-cell responses induced analyzing the bi-functionality of the CD8^+^T-cells in relation to their dual capacity to degranulate (measured as CD107_a/b_^+^ cells) and produce IFN-γ in splenocytes stimulated with B8R. Figure 5a shows the percentage of B8R specific CD107_a/b_^+^/IFNγ^+^ CD8^+^ T-cells that, for the MVA∆C12L/∆A44L-A46R vector, was over two-fold higher compared to MVAwt (*p <* 0.05).

We also analyzed the distribution and modulation of memory subpopulations involved, which were classified according to the combination of CD44 (extracellular matrix receptor, upregulated after T-cell activation) and CD62L (L-selectin, homing to LNs) markers as follows: “early-memory or T stem-cells memory” (T_SCM_; CD44L^−^/CD62L^+^) [52,53], T central memory (T_CM_; CD44^+^/CD62L^+^), T effector memory (T_EM_; CD44^+^/CD62L^−^), and T terminal effector memory (T_TEM_; CD44^−^/CD62L^−^). Figure 5b shows that bi-functional CD8^+^ T-cells (CD107_a/b_^+^/IFN-γ^+^) were, as expected, nearly only present inside the T_EM_ subpopulation, which showed significant increase of MVA∆A44L-A46R and MVA∆C12L/∆A44L-A46R *vs.* MVAwt (*p <* 0.001).

**Figure 6:**
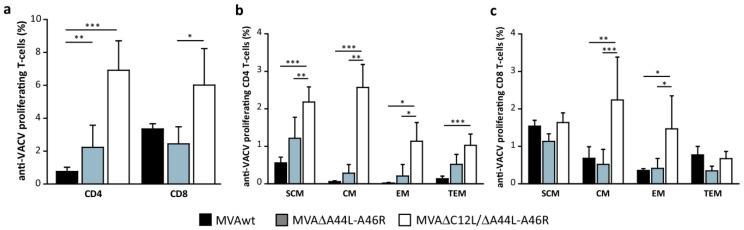
Analysis of the proliferation capacity of memory T-cells. (**a**) Splenocytes from MVAwt, MVA∆A44L-A46R or MVA∆C12L/∆A44L-A46R immunized mice were labeled with CFSE, stimulated with UV-inactivated VACV and stained with CD4 or CD8, CD44 and CD62L. Percentage of specific proliferating CFSE_low_ T-cells was determined by flow-cytometry. Cells were classified in four memory subpopulations (as detailed in Figure 5b), corresponding to CD4^+^ (**b**) or CD8^+^ (**c**) T-cells. Data are representative of three independent experiments. Statistical analysis performed by one-way ANOVA and Bonferroni’s post-test. Significant differences: * *p <* 0.05, ** *p <* 0.01, *** *p <* 0.001.

We also evaluated the specific proliferative potential of T-cells with CFSE-based proliferation assays as detailed in Materials and Methods. We found significant higher proliferation levels of anti-VACV T-cells (in CD4^+^ and CD8^+^ T-cells) in mice immunized with MVA∆C12L/∆A44L-A46R (Figure 6a). When we analyzed the memory subpopulations implicated in the specific proliferation observed (Figure 6b,c), the higher differences between MVA∆C12L/∆A44L-A46R *vs.* MVAwt and MVA∆A44L-A46R were found in the T_SCM_ (*p <* 0.001 and *p <* 0.01, respectively), T_CM_ (*p <* 0.001 and *p <* 0.01, respectively) and T_EM_ (*p <* 0.05) subpopulations inside the CD4^+^ T-cell compartment, and in the T_CM_ (*p <* 0.01 and *p <* 0.001, respectively) and T_EM_ (*p <* 0.05 *vs.* MVAwt) within CD8^+^ T-cells.

These results demonstrate that during the memory phase of the response, deletion of the *A44L-A46R* segment in combination with *C12L* (MVA∆C12L/∆A44L-A46R) generated an adaptive T-cell response of higher quality in relation to its poly-functionality and proliferative potential compared to MVAwt.

**Figure 7:**
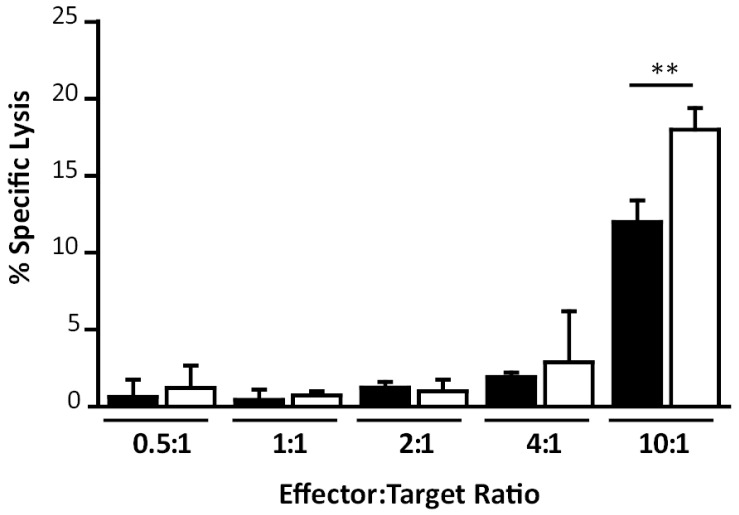
Study of *in vitro* T-cell specific killing activity. Specific cytotoxicity assay against B8R-pulsed cells was performed as described in Materials and Methods. Data are expressed as mean + SD of triplicate determinations and represent two independent experiments. Significant differences between MVAwt (black) and MVA∆C12L/∆A44L-A46R (white) are shown. One-way ANOVA and Bonferroni’s post-test. ** *p <* 0.01.

Finally after the results described above we considered relevant to evaluate the functionality of the CD8^+^ T-cell responses generated, analyzing the specific killing capacity of T-cells in mice immunized with MVAwt in comparison with MVA∆C12L/∆A44L-A46R. We performed an *in vitro* cytotoxicity assay, evaluating specific lysis against target cells pulsed with B8R peptide as described in Materials and Methods (Figure 7). It can be seen that cells from MVAΔC12L/ΔA44L-A46R immunized mice showed significant higher cytoxicity capacity compared to cells from MVAwt immunized mice (*p <* 0.01) (at a relation effector:target cells of 10:1), indicating that CD8^+^ T-cells from MVAΔC12L/ΔA44L-A46R immunized mice showed a higher functionality compare to those from MVAwt.

### 3.6. Pattern of Cytokines Induced during Innate Immune Response by MVA∆C12L/∆A44L-A46R

**Figure 8:**
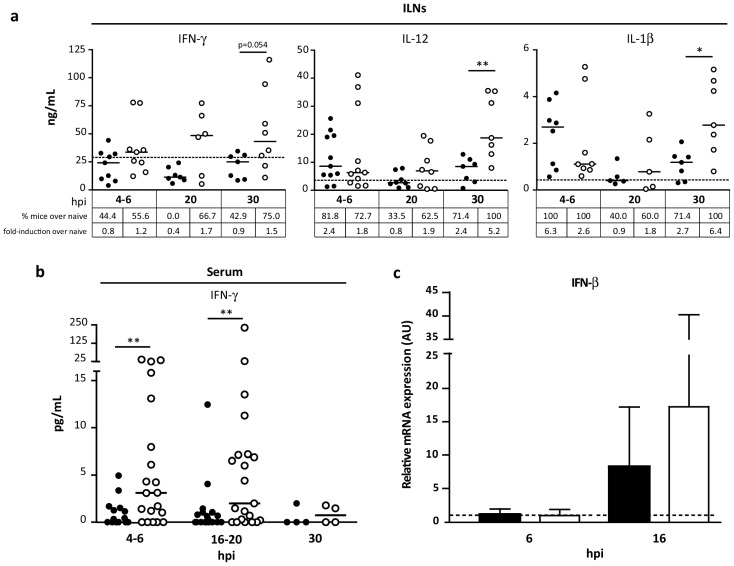
Analysis of the innate immune response *in vivo*. C57Bl/6 mice were i.m. immunized with 2 × 10^7^ PFU of MVAwt (black) or MVA∆C12/∆A44L-A46R (white) and at indicated hours post-inoculation (hpi) were sacrificed and samples processed as explained in Materials and Methods. Pro-inflammatory mediators were quantified in homogenates of ILNs (**a**) and serum (**b**) by ELISA. Lines represent median values; dots, individual mice and dotted line, naïve median. (**c**) At six and 16 hpi, six mice per group were sacrificed and RNA was extracted from ILNs. IFN-β mRNA was quantified by qPCR (normalized to β-actin) and relativized to naïve mice group (dotted line). Mann-Whitney test for statistical analysis (* *p <* 0.05, ** *p <* 0.01) was performed. Data are representative of two independent experiments.

The experiments described above showed that MVA∆C12L/∆A44L-A46R induced adaptive T-cell responses of higher magnitude and quality *vs.* MVAwt. Consequently, we analyzed the cytokine pattern of the innate immune response elicited *in vivo* by this vector compared to MVAwt. Groups of four to six C57Bl/6 mice immunized with MVAwt or MVA∆C12L/∆A44L-A46R were sacrificed at different time points (4–6, 16–20 and 30 hpi), and diverse pro-inflammatory mediators were evaluated in homogenates of ILNs (Figure 8a) and serum (Figure 8b). In ILNs animals inoculated with MVA∆C12L/∆A44L-A46R showed an increase in the levels of IFN-γ (*p =* 0.054), and importantly significant higher levels of IL-12 (*p <* 0.01) and IL-1β (*p <* 0.05) (Figure 8a) compared to MVAwt. We also analyzed the percentages of mice whose responses overcame the naïve median for each cytokine and the fold induction in each group, in relation to the naïve group. As observed, in the tables depicted below of each cytokine-graph, except for the shorter time-point for IL-12 and IL-1β, the MVA∆C12L/∆A44L-A46R induced higher percentages of mice with responses over the naïve value and also superior fold-inductions *vs.* MVAwt vector for all cases.

In sera we only found detectable values for IFN-γ, being higher in mice inoculated with MVA∆C12L/∆A44L-A46R *vs.* MVAwt at 4–6 hpi (*p <* 0.01) and 16–20 hpi (*p <* 0.05) (Figure 8b).

Immunization with MVA involves a host anti-viral response that triggers IRF3/IRF7 pathways [54,55], thus we also evaluated the production of IFN-β mRNA by qPCR. At 6 hpi, there was no difference between mice from both groups with respect to IFN-β mRNA levels in naïve mice (Figure 8c). However, at 16 hpi we found a higher expression of IFN-β in mice immunized with MVA∆C12L/∆A44L-A46R *vs.* MVAwt (although not significant). Even more we observed that, whereas the MVAwt group only two out of five mice showed a 10-fold increase in relation to naïve, four out of six mice reached this increment in the MVA∆C12L/∆A44L-A46R group and notably, two of them overcame the naïve group for more than 20-fold.

## 4. Discussion

Due to the relevance that MVA vectors currently have, there is a substantial need to improve the vector immunogenicity potential as many MVA-based vaccines are presently being developed for use against a number of prominent infectious diseases including AIDS, Malaria, and Tuberculosis, as well as human cancers [56]. Earlier studies demonstrated that it is feasible to improve the immunogenicity of MVA and generate better immune responses after deletion of specific viral-genes with functions related to evasion of the host immune responses [20,21,22,23,24]. In the present study, we have generated and characterized an MVA vector involving the deletion of the *A44L* and *A46R* genes (MVA∆A44L-A46R), and considering that the *C12L* and *A46R* gene products have inter-related functions, we also constructed another MVA including the deletion of this third gene too (MVA∆C12L/∆A44L-A46R).

It must be noticed that due to the strategy used in the deletion of the *A44L* and *A46R* genes, the *A45R* gene located between both genes was also eliminated. However, the product of this last gene has been previously described as an inactive superoxide dismutase-like protein carrying a deletion of 12 nucleotides [4,38]; therefore, taking this into account we discarded any possible effect on MVA due to the absence of the *A45R* gene*.* A significant point addressed in this study was to demonstrate that the absence of selected VACV genes resulted in a lack or at least significant decrease in the specific biological function associated with evasion of the host immune response. For this, we evaluated the effect of A46 absence by quantifying key inflammation markers produced after THP-1 infection. Previous studies have demonstrated that production of a mature IL-1β after MVA infection requires a crosstalk between TLR2-MyD88 and NALP3 inflammasome [57]. Therefore, considering that A46 interacts with MyD88 [25], the finding of a rapid increase in IL-1β in supernatants infected with MVA∆A44L-A46R or MVA∆C12L/∆A44L-A46R vectors compared to MVAwt, was indicative of absence of the A46 functionality in these MVAs. In relation to the A44 biological function, to our concern, and except only for one report in which it has been described the 3β-HSD activity for many Vaccinia strains including MVA [36], this is the first report in which the presence of the enzyme activity has been demonstrated in the MVAwt as well as its absence after deleting the *A44L* gene. Regarding the *C12L* biological activity, we demonstrated it in the MVA∆C12L/∆A44L-A46R, as we previously did with the MVA∆C12L vector [20], with significant increase of IFN-γ secretion in the bioassay after the removal of this gene.

Immunogenicity studies showed that CD8^+^ and CD4^+^ T-cell responses against VACV epitopes resulted in significant increase at 7 dpi. Importantly, during the memory phase (45 dpi), lymphocytes from animals immunized with deleted MVAs also showed anti-VACV cellular responses of higher magnitude compared to MVAwt (for IFN-γ, IL-2 and TNF-α production). The analysis of the cellular response quality in terms of the capacity of the CD8^+^ T-cells to simultaneously degranulate (CD107_a/b_) and secrete IFN-γ, showed that MVA∆C12L/∆A44L-A46R generated higher levels of B8R specific CD107_a/b_^+^/IFNγ^+^ CD8^+^T-cells in relation to MVAwt. Importantly, this T-cell bi-functionality was previously associated with improved viral suppression activities of CD8^+^ T-cells [58]. A desirable property for a vaccine vector is the induction of memory T-cells with proliferative potential. Notably, we detected significantly higher proliferation levels of anti-VACV T-cells (both CD4^+^ and CD8^+^ T-cells) after immunization with MVA∆C12L/∆A44L-A46R. It is important to remark, that we also found higher cytotoxicity capacity in splenocytes of mice immunized by this vector in comparison to MVAwt.

Previous studies have analyzed the immunomodulatory characteristics of the *A46R* gene, first defining that A46 impairs TLR-signaling by targeting the TIR domain of MyD88, Mal, TRIF and TRAM adaptors, disrupting the interaction with the receptor and consequently diminishing the production of pro-inflammatory cytokines as well as type I IFNs [25,26]. Moreover, it has also been shown that A46 contributes to virulence in VACV infections since the VACV *A46R* deletion mutant was attenuated in a murine intranasal model [26]. However, data analyzing the consequences of its deletion on poxvirus vaccine immunogenicity are somehow contradictory. One study [32] showed that deleting *A46R* from NYVAC, increased the inflammatory cytokine response in infected human macrophages, and also improved antigen immunogenicity after DNA-NYVAC vaccination. However, other authors that employed the MVA-BAC system for gene deletion reported that absence of the *A46R* or *A44L* genes did not generate an improvement in T-cell responses against epitopes of the vector [34]. More recently, the same authors reported that cluster gene deletion from MVA (including deletion of *B15R*, *A46R*, *A44L*, among others) also failed to improve acute or memory CD8^+^ T-cell frequencies against any antigen tested [33]. Different methodological factors may account for these discrepancies, like the procedures for the generation of the deleted MVAs, inoculation routes and viral doses applied. However, undoubtedly important features to be evaluated in order to determine if a viral gene deletion generates improvements in vector immunogenicity are the verification that the attributed biological function of the gene is no longer present and an *in vivo* analysis of the innate responses induced by the new modified vector. Both issues were directly demonstrated in our study for the three genes selected (*A46R*, *A44L*, and *C12L*) and one of them (innate responses) was indirectly corroborated by Perdiguero *et al.* [32]. However, none of the two previous studies involving MVA-BAC vectors that include deletions of the *A44L*, *A46R*, and *C12L* genes [33,34] showed any results demonstrating that the new MVAs induced changes either in the innate immune response of the host or in the key signaling pathway on which the selected gene was acting.

The primary adaptive immune response to most pathogens and vaccines is initiated in regional LNs draining peripheral sites of antigen exposure [59]. But surprisingly, none of those previous studies analyzed the innate responses that occur in draining LNs to the site of immunization. Those studies analyzed innate responses *in vitro*, employing human or murine macrophage cell-lines [60], or primary human monocytes [61]. Moreover, in one recent paper, which analyzed the *in vivo* innate responses against MVA, serum cytokines were analyzed after intravenous inoculation, which is not a usual immunization route [62]. Even more, many other studies related with deleted MVAs [18,22,33,34] did not perform any assay to characterize the innate response induced by the deleted vector. The results from our experiments demonstrated that at short times post-immunization (4 to 30 hpi) MVA∆C12L/∆A44L-A46R induced higher levels of cytokines with a determining role in the generation of T-cell immune responses such as IL-12 and IFN-γ [63,64,65]. Even more, MVA∆C12L/∆A44L-A46R also induced higher levels of IL-1β, a cytokine with reported effects to enhance CD4^+^ T-cells [66], and antigen-specific CD8^+^ T-cell responses [67]. In addition, higher levels of IFN-β mRNA were induced by the MVA∆C12L/∆A44L-A46R. This antiviral cytokine is also considered as an important “third signal” with capacity to shape effector and memory T-cell pools [68]. Indeed, previous studies demonstrated that production of type I IFN played a pivotal role in innate and adaptive immune responses to VACV infection [69] and also that IFN-β can promote the expansion of MVA-induced CD8^+^ T cells [70].

## 5. Conclusions

This study describes for the first time that simultaneous deletion from the MVA-backbone of VACV genes *A44L*, *A46R* and *C12L* (MVA∆C12L/∆A44L-A46R) was translated into a significant enhancement of CD4^+^ and CD8^+^ T-cell adaptive responses, and importantly major quality T-cell characteristics were improved. Absence of the biological functions of the deleted genes was demonstrated by specific assays, and after *in vivo* experiments we found that the deletion of the selected genes from MVA genome generated an innate response in the regional LNs to the site of immunization characterized by an increase in IFN-γ, IL-12, and also IL-1β and IFN-β.

## Supplementary Materials

The following are available online www.mdpi.com/1999-4915/8/5/139/s1, Figure S1: *In vitro* characterization of MVA∆A44L-A46R and MVA∆C12L/∆A44L-A46R, Figure S2: Quantification of IgG, IgG1 and IgG2a levels in serum from immunized mice.

## Abbreviations

The following abbreviations are used in this manuscript:

CEF	chicken embryo fibroblasts
ConA	Concanavalin A
CVA	Chorioallantois vaccinia virus Ankara
DMEM	Dulbecco’s Modified Eagle’s Medium
DMSO	dimethylsulfoxide
dpi	days post-infection
FBS	fetal bovineserum
GUS	β-glucuronidase enzyme
hpi	hours post-infection
ICS	Intracellular cytokine staining
MVA	Modified Vaccinia Ankara Virus
moi	multiplicity of infection
NYVAC	New York Vaccinia virus
ORF	open reading frames
PFU	plaque former units
PMA	phorbol-12-myristate-13-acetate
SD	Standard deviation
SFU	Spot-forming unit
SPF	specific pathogen free
T_CM_	T-cell Central Memory
T_EM_	T-cell Effector Memory
T_SCM_	T-cell Stem Cell Memory
T_TEM_	T-cell Terminally Effector Memory
TIR	Toll/interleukin-1 receptor
TLR	Toll Like Receptor
VACV	Vaccinia virus
WR	Western Reserve strain
3β-HSD	3β-hydroxysteroid-dehydrogenase/∆5-∆4 isomerase
