# Supplementary material for: Deletion of A44L, A46R and C12L Vaccinia Virus Genes from the MVA Genome Improved the Vector Immunogenicity by Modifying the Innate Immune Response Generating Enhanced and Optimized Specific T-Cell Responses
Source: Viruses. 2016 May 18;8(5):139. doi: 10.3390/v8050139 (PMC4885094; doi:10.3390/v8050139)
Supplement: Supplementary file 1 [file viruses-08-00139-s001.pdf]

Supplementary Materials: Deletion of *A44L*, *A46R* and *C12L* Vaccinia Virus Genes from the MVA Genome Improved the Vector Immunogenicity by Modifying the Innate Immune Response Generating Enhanced and Optimized Specific T-cell Responses

María Pía Holgado, Juliana Falivene, Cynthia Maeto, Micaela Amigo, María Fernanda Pascutti, María Belén Vecchione, Andrea Bruttomesso, Gabriela Calamante, María Paula del Médico-Zajac and María Magdalena Gherardi

Materials and Methods

2.1. PCR of Deleted Genes

In order to confirm *C12L* gene deletion, a set of primers that flank the complete sequence of the gene were used (C12L-l-F 5′-GAATTCGTTGTTTACTCAAAACG-3′; C12L-r-R 5′-GAGCTCCTATA ATGATTATATAG-3′). The expected amplification product, if the gene is present, is of approximately 1000 pairs of base. The absence of amplification product indicates that the gene was replaced by the reporter gene cassette (*lacZ* gene, expresses the beta galactosidase enzyme). In the case of the deletion of *A44L-A46R* segment, the forward primer hybridizes in the *A45R* internal gene (A45R-F 5′ TAATAGACCACGATAATATC-3′) and the reverse primer in the flanking region of *A46R* gene (A46R-r-R 5′-AGAATTCGCCCACATAAATGCGTTGGAG-3′). The expected amplification product, if the genes are present, is of approximately 1400 paires of base and absence of amplification product indicates the insertion of the reporter gene cassette (*uidA* gene, expresses the glucuronidase enzyme). Amplification of HA gene was performed as amplification control. Primers used were: HA1 5′-TCCCCGCGGATGACACGATTACCAATA-3′ and HA4 5′-GGGG TACCAGAATATTGCCACGGCCG-3′, and the expected amplification product is of approximately 890 pairs of base.

2.2. Immunofluorescence

THP-1 cells were differentiated into THP-1–derived macrophage in glass coverslips and infected with different MVAs (moi = 5), or mock infected. After 24 hpi cells were fixed and permeabilized with paraformaldehyde and methanol respectively, and subsequently incubated with polyclonal rabbit anti-VACV antibody and secondary antibody (Alexa-Fluor 594 anti-rabbit immunoglobulin, Jackson ImmunoResearch). Finally, coverslips were mounted on glass slides using DAPI-FluoromountG (Southern Biotech). Immunofluorescence images were acquired with a Nikon Eclipse fluorescence microscope using a CFI Plan Fluor 60-1.25 oil immersion objective. Images were analyzed using the NIS-Elements Software.

2.3. ELISA of Antibodies against VACV

The presence of antibodies against VACV in serum samples was determined by ELISA as described by Ramirez *et al.* [41] coating the plate with UV inactivated VACV at a final concentration of 5 μg/mL. Peroxidase-conjugated goat anti-mouse IgG (Jackson Immuno Research, Baltimore), IgG1 and IgG2a (Santa Cruz Biotechnology, Dallas) were diluted 1/4000.

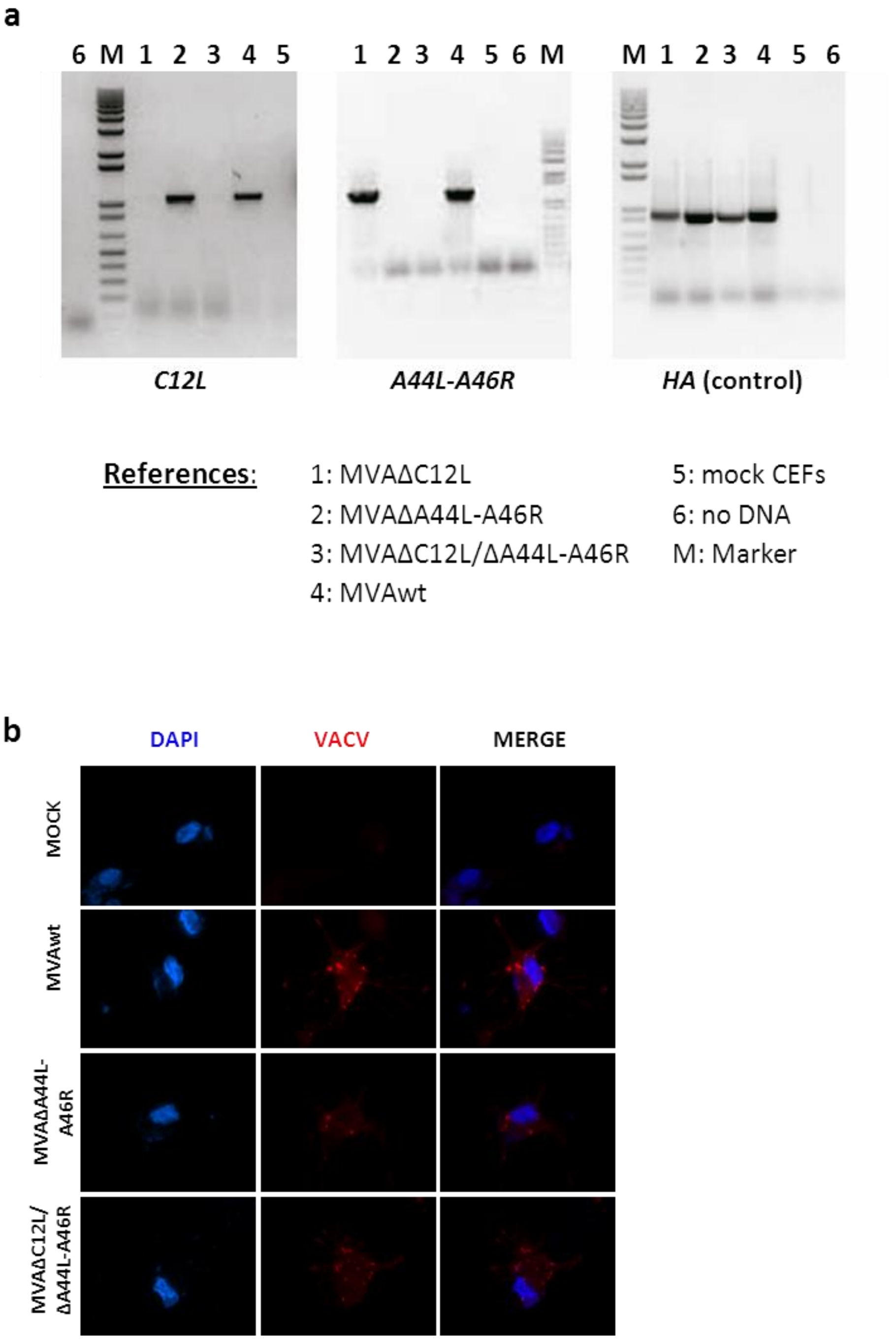

Figure S1. *In vitro* characterization of MVAΔA44L-A46R and MVAΔC12L/ΔA44L-A46R (**a**) PCR amplification of selected genes. DNA was extracted from uninfected CEFs (mock) or CEFs infected with the indicated MVAs and PCR was performed with specific primers; (**b**) Immunofluorescence analysis after MVA infection of THP-1 cells (moi = 5, 24 hpi). Images were taken at 60×.

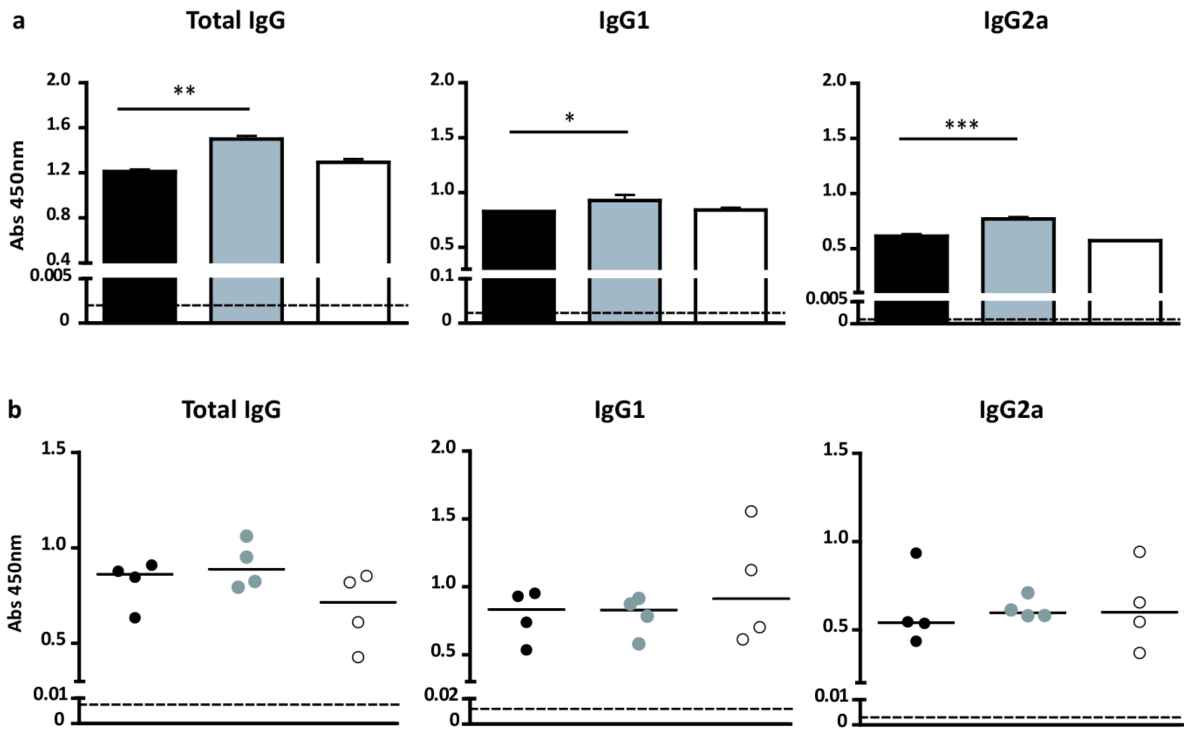

Figure S2. Quantification of IgG, IgG1 and IgG2a levels in serum from immunized mice. C57Bl/6 mice were immunized with MVAwt (black), MVAΔA44L-A46R (grey) or MVAΔC12L/ΔA44L-A46R, and 45 dpi sera were obtained. (**a**) Total IgG, IgG1 and IgG2a quantifications in sera of mice. Serum samples were pooled and diluted 1/1600. One-way ANOVA and Bonferroni's post test; (**b**) The same as in (**a**) but in this case serum samples were analyzed individually (each dot represents one animal). Sera dilutions employed were: for total IgG:1/3200, and for IgG1 and IgG2a: 1/1600. Mann-Whitney test. In all the graphs values found in sera from naïve mice are represented by the dotted line.

© 2016 by the authors; licensee MDPI, Basel, Switzerland. This article is an open access article distributed under the terms and conditions of the Creative Commons by Attribution (CC-BY) license (http://creativecommons.org/licenses/by/4.0/).
